# A Programmable Hybrid Energy Harvester: Leveraging Buckling and Magnetic Multistability

**DOI:** 10.3390/mi16040359

**Published:** 2025-03-21

**Authors:** Azam Arefi, Abhilash Sreekumar, Dimitrios Chronopoulos

**Affiliations:** Department of Mechanical Engineering & Mecha(tro)nic System Dynamics (LMSD), KU Leuven, 9000 Gent, Belgium; abhilash.sreekumar@kuleuven.be (A.S.); dimitrios.chronopoulos@kuleuven.be (D.C.)

**Keywords:** vibration energy harvesting, bistability, multistability, buckled beam, snap-through, magnetoelastic coupling, design optimization

## Abstract

Growing demands for self-powered, low-maintenance devices—especially in sensor networks, wearables, and the Internet of Things—have intensified interest in capturing ultra-low-frequency ambient vibrations. This paper introduces a hybrid energy harvester that combines elastic buckling with magnetically induced forces, enabling programmable transitions among monostable, bistable, and multistable regimes. By tuning three key parameters—buckling amplitude, magnet spacing, and polarity offset—the system’s potential energy landscape can be selectively shaped, allowing the depth and number of potential wells to be tailored for enhanced vibrational response and broadened operating bandwidths. An energy-based modeling framework implemented via an in-house MATLAB^®^ R2024B code is presented to characterize how these parameters govern well depths, barrier heights, and snap-through transitions, while an inverse design approach demonstrates the practical feasibility of matching industrially relevant target force–displacement profiles within a constrained design space. Although the present work focuses on systematically mapping the static potential landscape, these insights form a crucial foundation for subsequent dynamic analyses and prototype validation, paving the way for advanced investigations into basins of attraction, chaotic transitions, and time-domain power output. The proposed architecture demonstrates modularity and tunability, holding promise for low-frequency energy harvesting, adaptive vibration isolation, and other nonlinear applications requiring reconfigurable mechanical stability.

## 1. Introduction

Energy harvesting from ambient resources such as thermal gradients, electromagnetic waves, vibrations, and body movements provides a sustainable means of minimizing environmental impact while ensuring long-term energy availability [1]. This approach enables the creation of self-sustaining systems for low-power applications—such as wireless sensors, wearables, and IoT devices—where traditional batteries often face limitations in size, maintenance, and overall viability. However, low-frequency vibrational energy remains difficult to harness efficiently, as linear harvesters exhibit narrow bandwidths and reduced efficacy at sub-resonance frequencies. Consequently, researchers have explored nonlinear strategies, notably multistable configurations that leverage snap-through, inter-well transitions, and tunable mechanical properties to enhance power extraction over broader operating ranges. Multistable harvesters exploit elastic bistability and controlled magnetic coupling to manipulate potential wells and energy barriers, thereby expanding the vibrational response domain [2]. Recent bistable devices have utilized snap-through motions to elevate strain rates and boost energy output [3,4], while specialized beam geometries have been devised to lower energy barriers or facilitate sustained inter-well oscillations [5,6,7,8]. Structural modifications, such as auxetic features combined with buckled beams, have further widened operational bandwidths and improved electrical conversion [9].

In parallel, magnet-assisted designs have introduced a key layer of tunability. Through magnetically induced forces, researchers have devised adaptive potential landscapes to achieve bistability or higher-order multistability [10], enabling large-amplitude oscillations that promote efficient energy extraction under diverse vibrational conditions [11,12,13]. Among piezoelectric materials, polyvinylidene fluoride (PVDF), has gained increasing attention as promising candidate for energy harvesting applications due to its flexibility, durability, and high electromechanical coupling [14,15].

Piezomagnetoelastic structures have additionally revealed bifurcations and chaotic regimes that enhance performance across broadband excitations [16,17]. More recent work has integrated adjustable magnetic forces into bi-directional harvesters for hybrid energy sources such as wind and vibration [18], while rolling-swing magnets have shown remarkable adaptability under varying excitations [19,20]. Hybrid systems that merge elastic bistability with magnetic coupling have thus achieved significant progress in overcoming the inherent challenges of low-frequency vibration harvesting. By capitalizing on post-buckling deformation and magnet-driven potential tuning, these designs maintain both robustness and flexibility across broader vibrational conditions. Several studies have demonstrated the effectiveness of hybrid approaches, including S-type beam generators for low-frequency settings [21] and adjustable energy barriers that promote frequent inter-well transitions [22].

Despite significant advances, existing hybrid energy harvesting systems are often constrained to fixed multistable regimes or rely on complex magnet arrays that are difficult to reconfigure. Considering the wide range of application of sensors and actuators with similar design, the main objective of the present work is introducing a novel, easily reconfigurable hybrid harvester that leverages multiple, precisely controlled magnet placements and strategic polarity inversion, combined with tunable beam buckling. Unlike previous designs that are limited to a single operational mode or require extensive reassembly to alter magnet configurations, this novel approach offers a modular architecture that can be systematically programmed to exhibit multistability. This grants significantly expanded control over the potential landscape, enabling transitions between monostable, bistable, and multistable states. This adaptability is particularly advantageous for diverse low-frequency environments, especially within the context of sensors, actuators, and small electronic devices [23]. While this study focuses on the static potential landscape, it lays the foundation for dynamic harvesting applications by demonstrating how beam geometry and magnet layout can be systematically tailored to optimize performance across a wide range of excitation conditions which is the plan for the future work.

The manuscript is organized as follows: Section 2 details the design strategy and operational principles of the harvester. Section 3 introduces the mathematical modeling and governing equations. Section 4 presents a rigorous analysis of the critical parameters—particularly those affecting energy barriers and stability transitions—demonstrating how various states (monostable, bistable, and multistable) can be induced. Finally, Section 5 summarizes the key findings, underscoring the harvester’s programmability and modular nature, and outlines future steps for dynamic testing and energy conversion validation.

## 2. Configuration and Key Concepts

**Figure 1:**
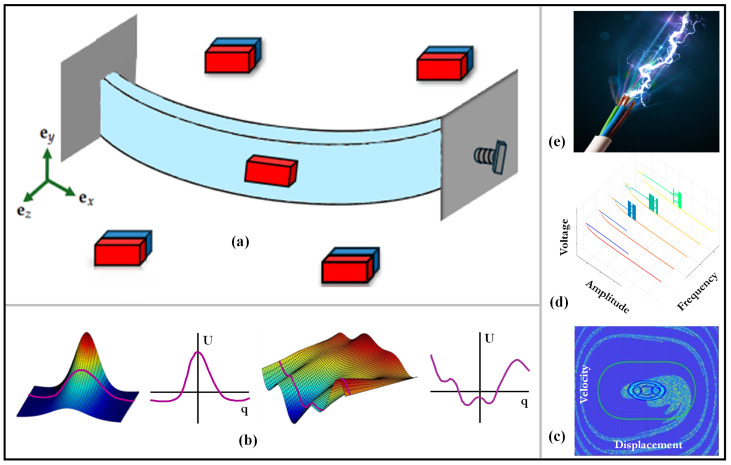
(**a**) Schematic representation of the harvester, (**b**) potential energy of the system and its variation with different design parameters (inspired from [24]), (**c**) Poincaré map and basins of attractions, (**d**) voltage bifurcation diagrams (generated with Stonehenge [25]), and (**e**) desired electrical power output.

The proposed energy harvester offers a modular and programmable design that combines elastic buckling with magnetically induced forces to achieve multiple stable regimes—ranging from monostable to multistable. Figure 1a provides an overview of the harvester’s core components. It features a double-cantilever beam of length *L*, width *b*, and thickness *h*, with Young’s modulus *E* and density ρ. The beam’s cross-sectional area is A=bh, and its bending moment of inertia is $I=\frac{bh^{3}}{12}$. The total beam mass is m=ρAL. The beam is buckled to a precise level $h_{0}$, introducing an elastic mechanism that can be exploited for bistability or multistability depending on other design parameters. A cubic “proof” magnet of height $h_{A}$ and mass $m_{A}$ is attached at the beam’s midpoint, serving a function analogous to the proof mass in conventional inertial harvesters.

Surrounding the beam are four “driving” magnets, each of height $h_{B}$ and mass $m_{B}$. These magnets are placed at a distance $d_{m}$ from the proof magnet and arranged in alternating polarities to ensure attraction from opposing sides. When $d_{m}$ is sufficiently small, the magnetic force can surpass the elastic restoring force, driving the beam through snap-through transitions and promoting inter-well oscillations. Such inter-well motion is widely recognized for enhancing energy harvesting by permitting larger-amplitude responses and higher strain rates.

Figure 1b schematically illustrates how variations in $h_{0}$, $d_{m}$ or magnet polarity reshape the potential energy landscape, thereby influencing intra-well and inter-well behavior. Basins of attraction, as depicted conceptually in Figure 1c, plays a pivotal role in determining whether the system settles in one potential well or transitions among multiple wells under external excitation. The voltage bifurcation diagrams in Figure 1d map how the harvester’s electrical output transitions among stable, periodic, or chaotic states as system parameters vary, highlighting optimal conditions for energy extraction. Lastly, Figure 1e outlines how these design features can be harnessed to maximize power output, underscoring the harvester’s adaptability and programmability in low-frequency energy harvesting applications.

## 3. Mathematical Formulation and Governing Equations

This section establishes the theoretical foundation for analyzing the harvester’s response to elastic and magnetic forces. First, the derivation of the beam’s elastic potential energy and its corresponding restoring force is presented, followed by the formulation of the magnetic potential energy based on dipole–dipole interactions. These expressions are then combined to obtain the net governing equations, which define the equilibrium conditions and pave the way for the parametric analyses discussed in subsequent sections.

### 3.1. Elastic Potential Energy and Force

The total elastic potential energy of the system $U^{el}$ is expressed as

(1) $$\begin{array}{cc} U^{el}= & \underset{\mathrm{bending}}{\underset{︸}{\frac{1}{2}EI\int_{0}^{L}{\left[w^{\prime \prime }\left(x,t\right)\right]}^{2}\;dx}}-\underset{\mathrm{axial}\;\mathrm{compression}}{\underset{︸}{\frac{1}{2}P\int_{0}^{L}{\left[w^{\prime }\left(x,t\right)\right]}^{2}\;dx}}\\ \; & +\underset{\mathrm{mid}-\mathrm{plane}\;\mathrm{stretching}}{\underset{︸}{\frac{1}{8L}EA{\left(\int_{0}^{L}{\left[w^{\prime }\left(x,t\right)\right]}^{2}\;dx\right)}^{2}}} \end{array}$$

where w(x,t) and *P* represent transverse displacement and axial compressive force, respectively. First and second partial derivatives with respect to *x*, i.e., $\frac{\partial }{\partial x}\left[\cdot \right]$ and $\frac{\partial^{2}}{\partial x^{2}}\left[\cdot \right]$ are concisely notated as ${\left[\cdot \right]}^{\prime }$ and ${\left[\cdot \right]}^{\prime \prime }$. Since the present study focuses on the mechanical response of the structure in the post-buckled state, the total deflection of the beam is approximated as [3,26,27]

(2) $$w\left(x,t\right)=h_{0}\phi \left(x\right)+\eta \left(t\right)\cdot \phi \left(x\right)=q\left(t\right)\cdot \phi \left(x\right),$$

which corresponds to the time-dependent deflection around the initial shape $h_{0}\phi \left(x\right)$ where $h_{0}$ is the initial midpoint buckling height and ϕ(x) is the first mode shape. q(t) denotes a generalized time function. For a double cantilever beam under axial compression, the first buckling mode shape and its corresponding buckling load are [28]

(3) $$\phi \left(x\right)=\frac{1}{2}\left(1-\cos\frac{2\pi x}{L}\right),\;\;P_{cr}=\frac{4\pi^{2}EI}{L^{2}}.$$

Substituting Equation (2) into Equation (1) yields

(4) $$\begin{array}{cc} U^{el}= & \frac{1}{2}\;\left[\int_{0}^{L}EI\phi^{\prime \prime 2}dx-P\int_{0}^{L}\phi^{\prime 2}dx\right]q{\left(t\right)}^{2}\;+\frac{1}{4}\left[\frac{EA}{2L}{\left(\int_{0}^{L}\phi^{\prime 2}\;dx\right)}^{2}\right]q{\left(t\right)}^{4}. \end{array}$$

Collecting the coefficients of $q{\left(t\right)}^{2}$, $q{\left(t\right)}^{4}$ and omitting the corresponding explicit time dependencies for ease of presentation, the above equation reads

(5) $$U^{el}=\frac{1}{2}k_{1}q^{2}+\frac{1}{4}k_{3}q^{4},$$

where $k_{1}$ and $k_{3}$ represent equivalent linear and nonlinear stiffnesses. Employing ϕ(x) from Equation (3) in Equation (4), one retrieves expressions for these equivalent stiffnesses.

(6) $$k_{1}=\frac{2\pi^{4}EI}{L^{3}}-\left(P_{cr}+\frac{\pi^{2}EAh_{0}^{2}}{4L^{2}}\right)\frac{\pi^{2}}{2L},\;\;k_{3}=\frac{\pi^{4}EA}{8L^{3}}.$$

The elastic restoring force is subsequently calculated

(7) $$F^{el}=-\frac{\partial U^{el}}{\partial q}=-\left(k_{1}q+k_{3}q^{3}\right).$$

This configuration achieves equilibrium ($F^{el}=0$) at the points $q_{eq}=\pm \sqrt{\frac{-k_{1}}{k_{3}}}$. It is to be noted that these states may be very different for scenarios involving magnetoelastic coupling.

### 3.2. Magnetic Potential Energy and Force

In dipole theory [29], magnetic potential energy is defined as $U^{m}=-B_{\mathrm{BA}}\cdot \mu_{A}$, where $B_{BA}$ is the magnetic flux density produced by dipole B on dipole A and is written as

(8) $$B_{BA}=-\frac{\mu_{0}}{4\pi }\nabla \left(\frac{\mu_{B}\cdot r_{\mathrm{BA}}}{∥r_{\mathrm{BA}}{∥}_{2}^{3}}\right).$$

**Figure 2:**
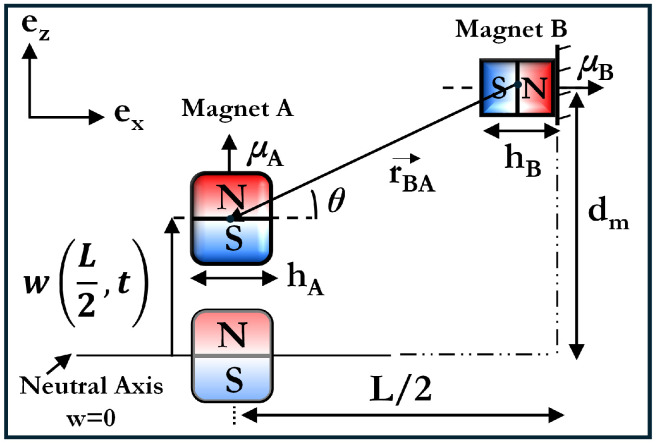
Schematic representation of magnetic force balance in the system.

The physical constant $\mu_{0}=4\pi \times 10^{-7}\;H/m$ contains the magnetic permeability of vacuum. The operators ∇(·) and ${∥\cdot ∥}_{2}$ respectively denote the vector gradient and Euclidean norm. The magnetic moment vectors of magnets A and B, i.e., $\mu_{A}$ and $\mu_{B}$ are separated by the position vector $r_{BA}$ as illustrated in Figure 2. These quantities are defined as follows

(9) $$\begin{array}{cc} \mu_{A} & =MV_{A}=Mh_{A}^{3}e_{z},\\ \mu_{B} & =MV_{B}=Mh_{B}^{3}e_{x},\\ r_{\mathrm{BA}} & =-r_{x}e_{x}-r_{z}e_{z}=-\frac{L}{2}e_{x}-\left(d_{m}-w\left(x,t\right)\right)e_{z}, \end{array}$$

where $e_{x}$, $e_{z}$ denote directional unit vectors. $M=\frac{Br}{\mu_{0}}$ is the magnitude of the magnetization vector, and $B_{r}$ is the residual magnetic flux density. $V_{A}$ and $V_{B}$ are the volumes of magnets A and B, respectively, which have been defined in terms of magnet A and B heights, i.e., $h_{A}$ and $h_{B}$, respectively. $d_{m}$ is the initial distance between the center of beam mid-magnet and surrounding magnet in the z-direction.

Combining Equations (8) and (9) and simplifying, one obtains

(10) $$U^{m}=-B_{\mathrm{BA}}\cdot \mu_{A}=\frac{\mu_{0}}{4\pi }\left(\frac{\mu_{A}\cdot \mu_{B}}{r^{3}}-3\frac{\left(\mu_{A}\cdot r_{\mathrm{BA}}\right)\left(\mu_{B}\cdot r_{\mathrm{BA}}\right)}{r^{5}}\right),$$

where $r=∥r_{\mathrm{BA}}{∥}_{2}$. Performing the necessary vector algebra, this is recast as

(11) $$U^{m}=-\frac{3\mu_{0}\left(M^{2}h_{A}^{3}h_{B}^{3}\right)}{4\pi r^{5}}\left[d_{m}-w\left(x,t\right)\right]\frac{L}{2}.$$

The component of the driving magnetic force of dipole B on dipole A in the z-direction can be derived from the magnetic potential energy by the following equation:

(12) $$F^{m}=-\frac{\partial U^{m}}{\partial r_{z}}=-\frac{3\mu_{0}\left(M^{2}h_{A}^{3}h_{B}^{3}\right)}{4\pi r^{4}}\left[1-5\cos^{2}\theta \right]\sin\theta$$

where $\cos\theta =\frac{r_{z}}{r}$ and $\sin\theta =\frac{r_{x}}{r}$. The trade-off between the magnetic and elastic forces determines the final mechanical response of the system. In such cases, equilibrium is governed by

(13) $$F_{net}=F^{el}-F^{m}=0$$

The equilibrium points are accordingly calculated by substituting Equations (7) and (12) into Equation (13). In the subsequent analysis, $h_{0}$, $d_{m}$, and other design parameters will be treated as variables to systematically tune the harvester’s stability states. To facilitate such design optimization, Table 1 summarizes the closed-form expressions for magnetoelastic potentials, forces, and their partial derivatives.

It is to be noted that, while this configuration does not explicitly accommodate piezoelectric patches, model order reduction strategies involving complexity reduction [30] and evaluating equivalent vibration parameters [31] ensure that the formalism developed in this work is sufficiently general and can be applied to more complex multilayer systems as well. The next section leverages these expressions in a parametric study, demonstrating how different parameter choices lead to monostable, bistable, or multistable configurations.

## 4. Results and Discussion

The energy harvesting capability of the proposed structure is fundamentally dictated by its equilibrium states, which govern its dynamic response under external excitation. In bistable and multistable systems, the nature of these states determines whether the system exhibits intra-well oscillations (confined within a single potential well) or inter-well transitions (snap-through between wells). Prior studies have established that inter-well motion can significantly enhance energy harvesting by inducing higher strain rates and greater mechanical-to-electrical conversion efficiency [32,33,34]. Therefore, designing an energy harvester with reconfigurable equilibrium states is crucial for optimizing its performance across a broad range of excitation conditions.

**Figure 3:**
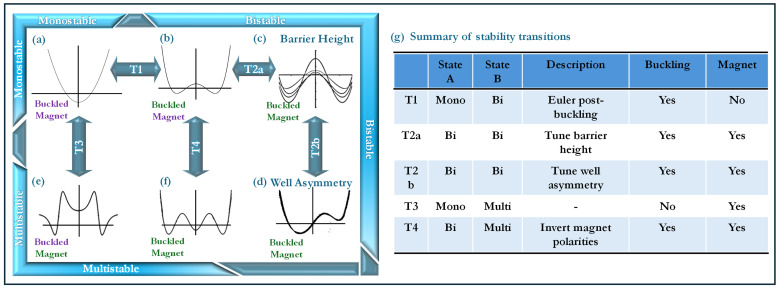
Schematic representation of stability transitions in the system. (**a**) Monostable state with a single equilibrium, (**b**) bistable state induced by buckling beyond the critical load, (**c**) barrier height modulation through magnetic interactions, (**d**) well asymmetry introduced by offsetting magnet placement, (**e**) multistable state obtained through additional magnetic tuning, (**f**) programmable multistability controlled by adjusting magnet polarity, and (**g**) properties of all Transitions (**T**) across stability states.

This multi-modality, as summarized in Figure 3g is primarily generated through

- *Buckling-induced bistability (T1):* where an initially monostable system transitions to a bistable configuration.
- *Magnetically induced multistability (T3):* enabling additional equilibrium states beyond classical buckling effects.
- *Magnet-assisted potential well tuning (T2a, T2b, T4):* allowing precise control over well depth and barrier height.

A bistable configuration, shown in Figure 3g, is achieved by introducing a compressive axial load that induces buckling in an initially monostable structure, as seen in Figure 3a. The degree of bistability is directly influenced by the level of buckling, which governs the depth and separation of the resulting potential wells. Further modifications to the energy landscape are possible through magnetically induced effects, allowing precise control over the system’s potential energy profile. The potential barrier height can be adjusted without significantly altering well locations by replacing the proof mass with a magnet of equivalent mass, as depicted in Figure 3c. This enables fine-tuning of the potential well depth while preserving the bistable nature of the harvester.

Beyond simple bistability, additional tuning mechanisms can introduce asymmetry into the potential energy profile, which can be achieved by strategically offsetting the stationary magnets on either side of the beam, as shown in Figure 3d. This asymmetry facilitates directional control of inter-well transitions, which is particularly useful for harvesting energy under variable excitation conditions. Furthermore, multistable behavior can be induced without relying on post-buckling effects, purely through controlled magnetic interactions, as illustrated in Figure 3e. The ability to manipulate the number and position of potential wells in this manner significantly enhances the adaptability of the system. Additionally, by inverting the polarities of the magnets on one side, more programmable multistable states can be obtained, further expanding the range of achievable equilibrium configurations, as seen in Figure 3f.

**Figure 4:**
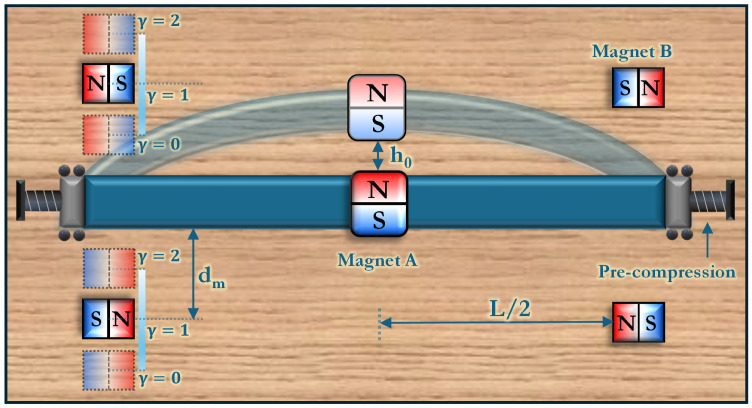
Schematic of the hybrid harvester, showing a buckled beam (with buckling level $h_{0}$) and strategically placed magnets. The magnet distance ($d_{m}$) and asymmetry parameter (γ) provide tunable control over the system’s potential landscape. The degree of buckling $h_{0}$ is controlled through generating pre-compression $P>P_{cr}$ in the beam.

In principle, a full performance characterization of an energy harvester involves analyzing basins of attraction, transient phase diagrams and Poincaré maps, and real-time power output. However, each distinct combination of the device’s structural parameters yields a unique potential energy topology, creating a vast design space. Consequently, this article focuses exclusively on mapping and shaping the potential energy landscape—establishing how to transition among monostable, bistable, and multistable states—and thereby laying a foundation for future work on orbit evolution, voltage generation, and power harvesting. To systematically investigate the sensitivity of these equilibrium states and transitions, a parametric study is conducted by varying the buckling level of the beam $h_{0}$, which determines the system’s mechanical bistability; the lateral distance $d_{m}$ of the stationary magnets from the beam, which influences the depth and shape of the potential wells; and the asymmetry parameter γ, which quantifies the degree of offset required to induce asymmetric behavior in the potential landscape. These parameters, illustrated in Figure 4, play a crucial role in governing the overall dynamics of the harvester. All other fixed material and geometric considerations are detailed in Table 2. The following sections detail how each parameter shapes the system’s steady state equilibria, setting the stage for a more comprehensive future analysis of basins of attraction, power output, and real-time orbit evolution.

### 4.1. T1: Monostable to Bistable

**Figure 5:**
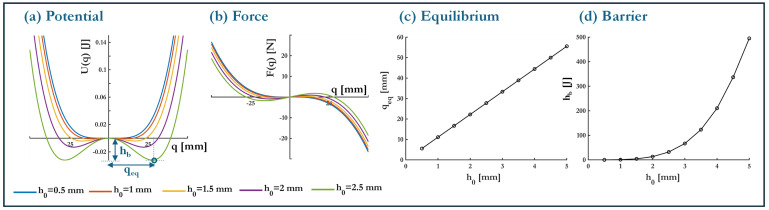
Effect of buckling level $h_{0}$ on bistability. (**a**) Potential energy curves, (**b**) force-displacement response, (**c**) equilibrium positions $q_{eq}$ as a function of $h_{0}$, and (**d**) potential barrier height $h_{b}$ as a function of $h_{0}$.

Mechanical bistability in a buckled beam is induced when the applied compressive load *P* exceeds the critical Euler buckling load $P_{cr}$ (Equation (3)), leading to bifurcation, transitioning from a monostable state to the formation of two stable equilibrium positions separated by a potential barrier (see transition from state (a) to (b) in Figure 3). The degree of bistability, characterized by the well depth and barrier height, is dictated by the extent to which *P* surpasses $P_{cr}$. This section investigates the relationship between the buckling level $h_{0}$ and the resulting bistability, with key results illustrated in Figure 5.

The potential energy landscapes, shown in Figure 5a, as computed at $n_{q}=2500$ points in the range q∈[−30,30] mm, depict the evolution of the system’s stability profile for various buckling levels, specifically $h_{0}=\left[0.5,1,1.5,2,2.5\right]$ mm. These potential curves exhibit horizontal symmetry, allowing full characterization using the equilibrium displacement $q_{eq}=\sqrt{\frac{-k_{1}}{k_{3}}}$, where $k_{1}$ and $k_{3}$ are the linear and cubic stiffness coefficients depicted in Equation (6). The potential barrier height, which quantifies the energy required for inter-well transitions, is given by $h_{b}=U\left(0\right)-U\left(q_{eq}\right)$. As evident in Figure 5a, increasing $h_{0}$ results in deeper potential wells and a larger energy barrier, enhancing the bistable nature of the system. The corresponding force-displacement curves, presented in Figure 5b, correspond to the elastic restoring force $F^{el}$ in Equation (7) and highlight the emergence of negative stiffness, an essential feature for facilitating snap-through motion. Notably, at higher buckling levels—particularly $h_{0}=2.5$ mm—the system exhibits a pronounced negative stiffness region, further reinforcing its bistable characteristics.

The equilibrium displacement $q_{eq}$ as a function of $h_{0}$, (see Figure 5c), is numerically determined using a hybrid CMAES–LBFGS optimization algorithm. Given the strongly multi-modal nature of the potential energy landscape and the need for precise extrema, this approach is chosen to balance global exploration and local refinement. The Covariance Matrix Adaptation Evolutionary Strategy (CMAES) mitigates dependence on initial conditions by efficiently exploring the search space [35], while Limited-memory BFGS (LBFGS) ensures fast and accurate convergence through gradient-based updates [36]. A linear relationship is observed, which aligns with theoretical expectations given that the linear stiffness component $k_{1}$ scales quadratically with $h_{0}$, i.e., $k_{1}\equiv f\left(P_{cr}\right)\propto h_{0}^{2}$ (see Equation (6)). Since $k_{1}$ is the only direct function of $h_{0}$, this quadratic dependence propagates to the system’s total potential energy, thus yielding a parabolic growth trend for the barrier height $h_{b}$ with respect to $h_{0}$ as shown in Figure 5d. This trend is consistent with prior studies on post-buckled beams, which emphasizes the critical role of the buckling level in defining the energy storage capacity of bistable systems [3]. Further investigation of these relationships can aid in optimizing transition dynamics, including orbit jumps during operation, which play a crucial role in energy extraction efficiency [37,38,39,40].

### 4.2. T2a: Bistable—Tuning the Potential Barrier Height via Magnetic Loading

The ability to fine-tune the potential energy landscape is crucial for optimizing the performance of bistable energy harvesters [22]. While $q_{eq}$ and $h_{b}$ are influenced by the buckling level $h_{0}$, finer control over these parameters is often required to enhance harvesting efficiency. In particular, increasing the barrier height $h_{b}$ plays a vital role in stabilizing inter-well transitions, which in turn maximizes the mechanical strain energy converted into electrical output [41].

**Figure 6:**
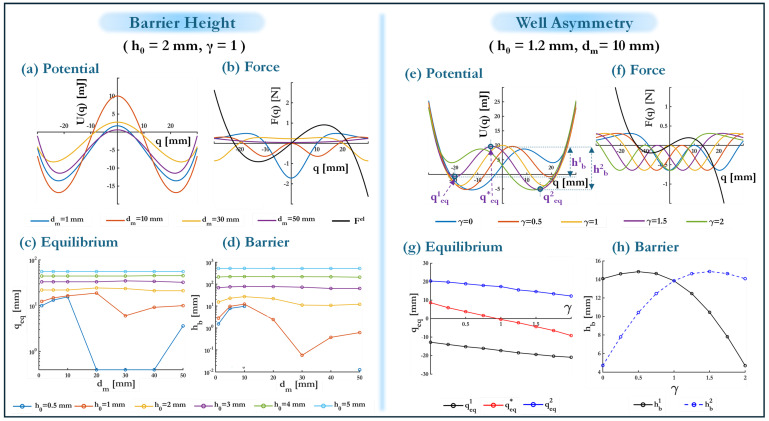
Effect of barrier height tuning and well asymmetry. (**a**) Potential energy curves for varying magnet distance $d_{m}$, (**b**) force-displacement response for different $d_{m}$ (colored and black lines denote $F^{m}$ and $F^{el}$, respectively), (**c**) equilibrium positions $q_{eq}$ as a function of $d_{m}$, (**d**) potential barrier height $h_{b}$ as a function of $d_{m}$, (**e**) potential energy curves for varying asymmetry parameter γ, (**f**) force-displacement response for different γ (colored and black lines denote $F^{m}$ and $F^{el}$, respectively), (**g**) equilibrium positions $q_{eq}$ as a function of γ, and (**h**) potential barrier heights $h_{b}^{1}$ and $h_{b}^{2}$ as a function of γ.

Replacing the proof mass with a magnet of equivalent mass $m_{A}$, oriented as illustrated in Figure 4, allows for barrier height modulation without significantly shifting the equilibrium positions (see transition from (b) to (c) in Figure 3). Since magnetic interactions are short-range forces that decay rapidly with distance, their effect is highly dependent on the perpendicular separation $d_{m}$ between the stationary magnets and the beam. The impact of $d_{m}$ on the system’s potential energy is analyzed for a fixed buckling level $h_{0}=2$ mm, with results displayed in Figure 6a.

A non-monotonic trend is observed in the barrier height as $d_{m}$ varies. Initially, increasing $d_{m}$ strengthens the interaction between the elastic restoring force and the magnet-induced forces, thereby increasing the potential barrier height. However, beyond an optimal distance—identified as $d_{m}=10$ mm—the influence of magnetic forces diminishes, leading to a reduction in $h_{b}$. At sufficiently large distances ($d_{m}=50$ mm), the system converges back to its purely elastic configuration, where the magnetic contribution is negligible. This trend can be explained by considering the balance of forces at the unstable equilibrium points (see Figure 6b), where inter-well transitions occur. When the magnets are placed too close to the beam, their attractive or repulsive forces $F_{m}$ dominate over the elastic restoring forces $F^{el}$, resulting in a weakened bistability due to the reduced ability of the beam to store elastic energy. Conversely, when $d_{m}$ is too large, the magnetic interactions become insufficient to significantly influence the energy landscape, thereby returning the system to its original buckling-dominated behavior.

To further examine the effect of magnetic tuning across different buckling levels $h_{0}$, the dependence of $h_{b}$ on $q_{eq}$ is analyzed in Figure 6c,d. At low buckling levels ($h_{0}=0.5$, 1 mm), the rate of change in $h_{b}$ with respect to $q_{eq}$ is substantially greater than that obtained by adjusting $h_{0}$ alone. This highlights the effectiveness of magnetic tuning at lower buckling amplitudes. However, at higher buckling levels ($h_{0}=3,4,5$ mm), the energy landscape is extensively dominated by elastic effects, making magnetic interactions less effective as a tuning mechanism. Notably, at $h_{0}=0.5$ mm, bistability is entirely lost for intermediate magnet distances ($d_{m}=20,30,40$ mm), as evidenced by the collapse of $q_{eq}$ to numerical precision limits in Figure 6c and zero or negative $h_{b}$ values in Figure 6d which are physically unrealistic. This suggests that for weakly buckled configurations, excessive magnetic tuning can induce undesired transitions back to an effectively monostable state.

### 4.3. T2b: Bistable—Introducing Well Asymmetry for Enhanced Energy Harvesting

Beyond barrier height tuning, introducing potential well asymmetry (see transition from state (c) to (d) in Figure 3) in spite of some disadvantages [42] has been shown to improve energy harvesting efficiency by biasing the system towards preferred transition directions, effectively increasing the probability of inter-well motion [43,44]. The proposed device accommodates this feature through controlling magnet positions, where the two stationary magnets are offset by an asymmetry parameter γ∈[0,2]. The modified distances of the magnets from the beam are given by

(14) $$d_{m}^{i}=d_{m}+{\left(-1\right)}^{i}\left(1-\gamma \right)d_{m},\;i=1,2,$$

where i=1,2 indexes the two sides of the beam. The symmetric case corresponds to γ=1, while deviations from this value induce asymmetry in the potential wells. The effect of γ on the equilibrium positions and energy barriers is illustrated in Figure 6e,f for a fixed $h_{0}=1.2$ mm and $d_{m}=10$ mm. For varying asymmetry parameters (γ=0,0.5,1,1.5,2), the stable equilibrium points $q_{eq}^{1}$ and $q_{eq}^{2}$ exhibit a mirror-antisymmetric behavior about γ=1. A similar symmetry is observed for the unstable equilibrium position $q_{eq}^{*}$. The potential barriers $h_{b}^{1}$ and $h_{b}^{2}$ also follow this mirrored trend but reach their maximum values at γ=0.5 and γ=1.5, respectively, before decreasing. This behavior arises from the non-uniform change in magnet spacing with γ, which leads to an imbalance in the stabilization of one potential well relative to the other. Notably, the shift of $q_{eq}^{*}$ away from zero introduces a directional bias in the system’s response, which may affect transition dynamics under external excitation.

**Figure 7:**
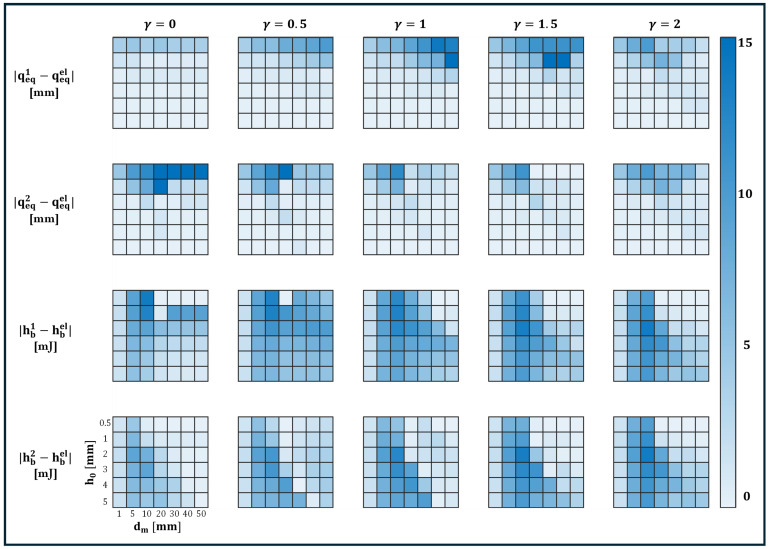
Heatmap representation of equilibrium position shifts and barrier height variations with respect to elastic buckling counterparts, as a function of magnet distance $d_{m}$ and asymmetry parameter γ. (Top two rows) Absolute differences in equilibrium positions $|q_{eq}^{1}-q_{eq}^{el}|$ and $|q_{eq}^{2}-q_{eq}^{el}|$. (Bottom two rows) Barrier height differences $|h_{b}^{1}-h_{b}^{el}|$ and $|h_{b}^{2}-h_{b}^{el}|$. Color intensity represents the magnitude of deviation, highlighting the sensitivity of the system to $d_{m}$, $h_{0}$ and γ.

To gain deeper insight into the system’s tuning sensitivity, the heatmaps in Figure 7 provide a comprehensive visualization of how the equilibrium positions $q_{eq}^{1}$, $q_{eq}^{2}$ and the potential barrier heights $h_{b}^{1}$, $h_{b}^{2}$ evolve as functions of the magnet distance $d_{m}$ and the asymmetry parameter γ. By representing the absolute deviations of these quantities relative to their *buckling-only* counterparts, i.e., $q_{eq}^{el}=\sqrt{\frac{-k_{1}}{k_{3}}}$ and $h_{b}^{el}=U^{el}\left(0\right)-U^{el}\left(q_{eq}^{el}\right)$, where the analytic expression for the elastic potential $U^{el}$ is provided in Equation (5), the heatmaps illustrate key nonlinear interactions and reveal threshold regions where stability transitions occur.

For symmetric configurations at γ=1, equilibrium deviations $q_{eq}^{1}$ and $q_{eq}^{2}$ (see first two rows of Figure 7) remain minimal across all $d_{m}$, preserving a balanced potential well structure. As asymmetry increases (γ=0.5,1.5), deviations become more pronounced, particularly in the range $d_{m}\approx$ 10–30 mm, where the interaction between elastic restoring forces and magnetic influences is strongest. At extreme asymmetry (γ=0,2), deviations further amplify, especially for lower $h_{0}$, indicating a strongly skewed potential landscape. However, for large $d_{m}$, the effects of asymmetry diminish, and equilibrium positions stabilize as elastic forces dominate.

Barrier height variations $\left(h_{b}^{1},h_{b}^{2}\right)$ (see rows 3–4 of Figure 7) exhibit a strong dependence on both $d_{m}$ and $h_{0}$. At moderate asymmetry (γ=0.5,1.5), the largest variations occur around $h_{0}\approx$ 2–3 mm and $d_{m}\approx$ 10–20 mm, suggesting that this range provides maximal tunability. For very small $h_{0}$, barrier heights become negligible, leading to a nearly monostable system. Conversely, at high $h_{0}$, the elastic energy dominates, reducing the impact of magnetic asymmetry. It should be noted that at γ=0,2, the model predicts $d_{m}=0$, which is non-physical as it implies the beam and magnet occupy the same position. However, this serves as an asymptotic limiting case that helps bound the parametric analysis. An optimal tuning regime emerges in the range 0.5≤γ≤1.5, $10\leq d_{m}\leq 30$ mm, and $h_{0}\approx$ 2–3 mm, where the equilibrium shifts and barrier height variations are maximized without destabilizing bistability. Outside this region, either asymmetry effects are too weak to be significant, or they excessively distort the potential landscape, reducing controllability.

### 4.4. T3: Monostable to Multistable

**Figure 8:**
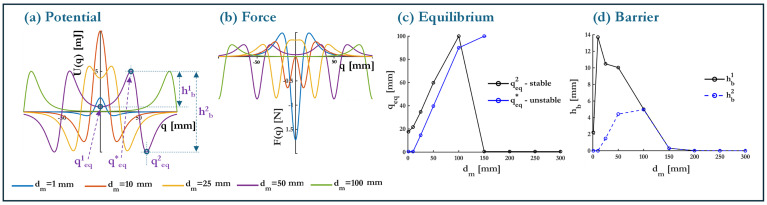
Effect of magnet distance $d_{m}$ on the transition from monostable to multistable configurations. (**a**) Potential energy curves for varying $d_{m}$, (**b**) force-displacement response with colored lines denoting $F^{m}$, (**c**) stable and unstable equilibrium positions $q_{eq}$ as a function of $d_{m}$, and (**d**) barrier heights $h_{b}^{1}$ and $h_{b}^{2}$ as a function of $d_{m}$.

The transition from a monostable to a multistable configuration (see transition from state (a) to (e) in Figure 3) introduces additional equilibrium states into the system, fundamentally altering its dynamic response. Unlike the previous bistable case, where only two potential wells existed, multistability arises through the controlled influence of magnetic interactions, which introduces additional energy minima in the potential landscape. The results presented in Figure 8 provide a detailed examination of how the system’s potential energy profile, force response, equilibrium configurations, and barrier heights evolve as a function of the magnet distance $d_{m}$.

The potential energy curves displayed in Figure 8a illustrate the transformation from a single-well monostable system (with $h_{0}=0$ mm) into a higher-order multistable configuration as $d_{m}$ increases. At very small magnet distances ($d_{m}$ = 1–10 mm), the system remains bistable, with two symmetric wells centered at $q_{eq}^{1}$ and $q_{eq}^{2}$, mimicking classical post-buckling elastic responses. However, as $d_{m}$ increases, additional potential wells emerge, leading to a more complex multi-well topology. For intermediate values of $d_{m}$ (e.g., 10–50 mm), the system exhibits three stable equilibrium positions, forming a triple-well potential structure. This suggests that within this range, the magnetic interactions are sufficiently strong to introduce new local minima without overwhelming the elastic restoring force.

A key feature of these potential profiles is that as $d_{m}$ increases beyond 50 mm, the additional wells begin to diminish, and the system gradually reverts toward a bistable or weakly multistable regime. This trend is indicative of a bounded region of effective multistability, where beyond a certain threshold, the magnetic interactions become too weak to sustain additional equilibria. The fact that multistability is not indefinitely scalable suggests that the influence of $d_{m}$ is highly nonlinear, requiring careful tuning to achieve an optimal number of equilibrium points. The force-displacement responses in Figure 8b provide further confirmation of this trend. At smaller $d_{m}$, the force curve exhibits two dominant restoring regions, characteristic of bistable behavior. As $d_{m}$ increases, the force response becomes progressively more complex, with additional equilibrium points forming at intermediate positions. These new equilibria correspond to the emerging potential wells observed in the energy curves. The presence of multiple force equilibrium crossings confirms that multistability is established through a balance of elastic and magnetic forces. However, at very large magnet separations, the force curve begins to smooth out, with the number of equilibrium points reducing once again, indicating a return to a simpler stability regime.

To systematically track the transition from bistability to multistability, the equilibrium configurations are analyzed in Figure 8c. The stable equilibrium position $q_{eq}^{2}$ ($q_{eq}^{1}=-q_{eq}^{2}$ in symmetric potential plots) and additional emergent equilibria are plotted as a function of $d_{m}$. At small $d_{m}$, only two dominant stable equilibria exist, corresponding to the conventional bistable case. As $d_{m}$ increases, additional stable positions appear, marking the formation of a multistable structure. The number of stable equilibria continues to increase up to an optimal $d_{m}$ range (approximately 100 mm), where the system exhibits the highest degree of multistability with 3 wells. Beyond this range, the additional equilibria begin to collapse, leading to a return to a simpler stability profile.

An important observation from Figure 8c is the behavior of the unstable equilibria, shown in blue, which are notably offset from q=0. These points serve as transition thresholds between neighboring stable wells, governing inter-well motion. As $d_{m}$ increases, the unstable equilibria shift outward, reaching a critical threshold at $d_{m}=150$ mm. Beyond this point, the smoothing effect causes these transition points to move beyond the relevant range, effectively limiting their influence on the system dynamics. This shift directly impacts the potential barrier heights $h_{b}^{1}$ and $h_{b}^{2}$ shown in Figure 8d as a function of $d_{m}$, revealing a distinct non-monotonic trend. At small magnet distances, the barrier heights are relatively large, reinforcing strong bistability with well-defined energy wells. As $d_{m}$ increases and multistability emerges, the barrier heights decrease, reaching a minimum at intermediate magnet distances. This reduction suggests that inter-well transitions become more energetically favorable, requiring less external excitation, which is advantageous for maximizing transition-driven energy harvesting. At very large magnet separations ($d_{m}>150$ mm), the system returns to a more conventional bistable regime where magnetic interactions weaken. This reinforces the observation that multistability is most effective within a specific intermediate range of $d_{m}$.

### 4.5. T4: Bistable to Multistable

**Figure 9:**
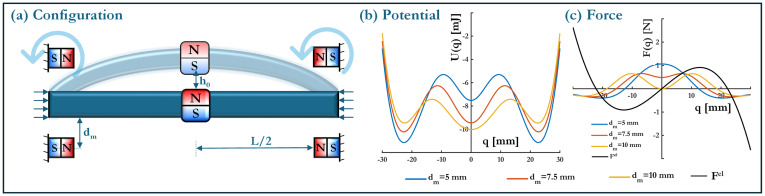
Transition from bistable to multistable configuration through top magnet polarity inversion. (**a**) Schematic of the modified system, (**b**) potential energy curves for varying $d_{m}$, and (**c**) force-displacement response illustrating the effect of the top magnet on system dynamics (colored and black lines denote $F^{m}$ and $F^{el}$, respectively).

The schematic in Figure 9a highlights the crucial polarity inversion in the top magnets, a key modification compared to the earlier cases. Unlike previous configurations, the top magnets now act as a repulsive force source, dynamically interacting with the beam’s deflection modes. By leveraging this additional degree of freedom, the system is capable of transitioning from a simple bistable configuration into a higher-order multistable regime. This design bridges the gap between the monostable to multistable transition, previously achieved solely by adjusting the stationary magnet distances, and the barrier height modulation, controlled via selective magnet positioning. The result is a programmable multistability framework, where stability states can be finely adjusted by a combination of buckling, magnetic asymmetry, and controlled repulsive forces (see transition from (b) to (f) in Figure 3).

The impact of this configuration on the potential energy landscape is shown in Figure 9b, where the potential curves are plotted for different values of $d_{m}$. Compared to the earlier bistable case, an additional local minimum emerges at $q_{eq}=0$ as $d_{m}$ is increased. This effectively merges the two key mechanisms previously analyzed: *(1) T3: multistability induction via magnet positioning*, and *(2) T2a: barrier height tuning via selective repulsive/attractive interactions*. This bridges the gap between structural buckling effects and magnetic energy modulation, offering a programmable and highly adaptable potential energy landscape. The ability to simultaneously adjust the number of potential wells and modify energy barriers opens new possibilities for controlling energy harvester dynamics. By carefully selecting $d_{m}$ and top magnet polarity, one can dictate whether the system operates in a high-barrier, low-frequency transition mode or a low-barrier, high-frequency inter-well motion regime. With this final transition from bistability to multistability, the system is now capable of exhibiting fully tunable stability states, ranging from simple bistable configurations to complex multi-well structures, as summarized in Table 3.

These recommended parameter ranges for $h_{0}$, $d_{m}$, γ are illustrative of the current geometry and material properties, rather than universal for all magneto-elastic harvesters. Different beam dimensions or magnet strengths may require re-tuning these values to achieve comparable performance. To bridge the gap between theory and application, the following section develops a design framework that matches a desired force–displacement profile under realistic engineering constraints.

### 4.6. Inverse Design for Optimizing Force-Displacement Response

In practical energy harvesting applications—ranging from structural health monitoring to self-powered sensors—the efficiency of energy conversion is highly dependent on the system’s ability to facilitate inter-well transitions under ambient excitations. A well-designed net force-displacement $\left(F_{net}-d\right)$ response is crucial in tuning these transitions, ensuring that the system efficiently captures and utilizes vibrational energy. This section formulates an inverse design framework to identify the optimal combination of design parameters $\mu_{\mathrm{opt}}\equiv {\left[h_{0},d_{m},\gamma \right]}_{\mathrm{opt}}$ that best replicates a target $\left(F_{net}^{*}-d\right)$ curve tailored for enhanced energy harvesting performance. The optimization problem minimizes the residual between the target and the device’s achievable force profiles, expressed as

(15) $$\mu_{\mathrm{opt}}\equiv {\left[h_{0},d_{m},\gamma \right]}_{\mathrm{opt}}=\left\{\begin{array}{c} arg\;min\;r\left(\mu \right)=\sqrt{\sum_{i=1}^{n_{q}}\frac{{\left(F_{net}^{*}\left(q_{i}\right)-F_{net}\left(\mu ,q_{i}\right)\right)}^{2}}{n_{q}}}\\ \mathrm{subject}\mathrm{to}:\\ 0\;\mathrm{mm}\leq h_{0}\leq 5\;\mathrm{mm}\\ 0\;\mathrm{mm}\leq d_{m}\leq 20\;\mathrm{mm}\\ 0.5\leq \gamma \leq 1.5 \end{array}\right..$$

These constraints ensure that the resulting design remains feasible, preventing excessive buckling, impractical magnet placements, or extreme asymmetry values that could hinder real-world physical realizability. The optimization leverages closed-form expressions for $F_{net}$ and its sensitivities $\partial F_{net}/\partial h_{0}$, $\partial F_{net}/\partial d_{m}$ and $\partial F_{net}/\partial \gamma$ (see Table 1) evaluated using a steepest gradient descent algorithm. A step size of $1\times 10^{-3}$, a relative residual convergence tolerance of $1\times 10^{-6}$, and an iteration limit of $1\times 10^{4}$ were employed to ensure numerical stability and convergence. Given the strongly multi-modal nature of the search space, a single run of gradient-based optimization could be highly dependent on the initial conditions. To mitigate this issue, the optimization was repeated 25 times with initial points sampled using a Latin Hypercube approach, ensuring broad coverage of the design space. The final design parameters are summarized in Table 4, where the estimated design parameters for three configurations *Only Magnet, Only Buckling* and *Magnet + Buckling* are presented. Each configuration represents a different strategy for achieving the desired force-displacement characteristics. The results correspond to the statistical mode of the obtained solutions, representing the most frequently occurring optimal configuration.

In the *Only Magnet* scenario, where elastic buckling is absent, the system relies heavily on magnet-induced asymmetry to introduce bistability by selecting a small magnet spacing $d_{m}=1$ mm and relatively high asymmetry of γ=1.5. However, the resulting $F_{net}-d$ curve does not adequately match the target, as magnetic forces alone are insufficient to create the desired nonlinearity. In contrast, the *Only Buckling* case, which excludes magnets, requires a higher buckling level of $h_{0}=2.35$ mm to achieve bistability. The absence of magnetic contributions limits the tunability of the potential wells, making it difficult to finely adjust barrier heights or well depths. While this configuration introduces appropriate mechanical nonlinearity, it lacks the flexibility needed for precise response shaping. The *Magnet + Buckling* approach indicates that a moderate buckling level $h_{0}=2.18$ mm combined with magnet induced local variations provides the best compromise between bistability strength and tunability.

**Figure 10:**
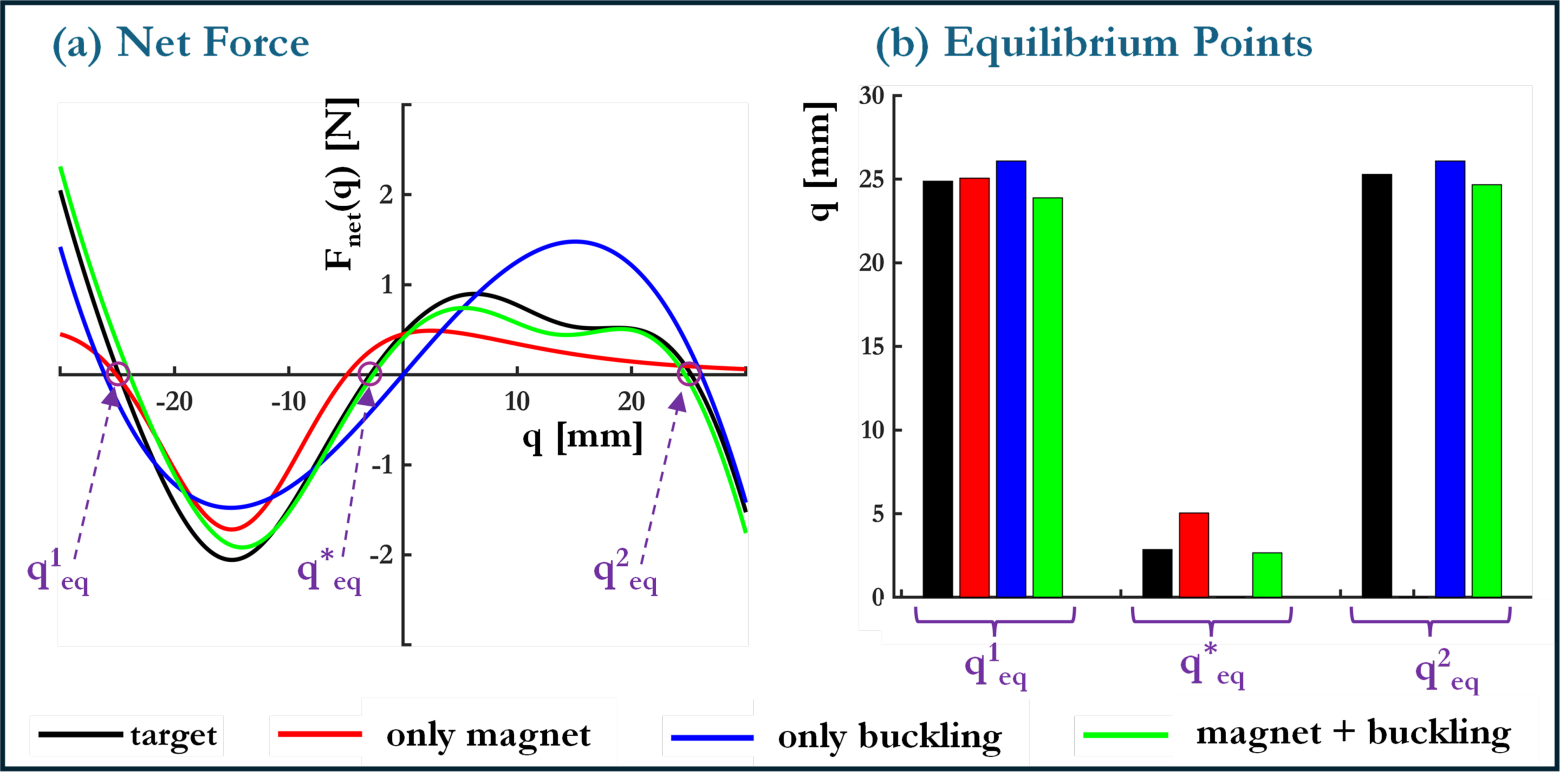
Optimized force-displacement response. (**a**) Net force comparison between the target and optimized configurations and (**b**) corresponding equilibrium position magnitudes for different design strategies.

Figure 10a compares the target and optimized force-displacement curves, showing that the *Magnet + Buckling* configuration achieves the closest match. The corresponding equilibrium positions, shown in Figure 10b, further illustrate the impact of tuning. The *Magnet + Buckling* case achieves well-defined stable and unstable equilibria, critical for enabling controlled inter-well transitions in bistable/multistable systems. It is to be noted that while the optimization framework effectively identifies feasible design parameters, the reliance on local gradient-based updates may still miss globally optimal solutions in highly nonconvex regions, even with rigorous sampling.

To summarize, the parametric exploration, together with the inverse design optimization, highlights the structure’s modular and programmable nature. It has been shown that the precise tuning of the beam’s buckling level, magnet spacing, and magnet polarity allows the system to span monostable, bistable, or multistable regimes, and that an inverse design approach can generate a target force–displacement profile on demand. In doing so, the results establish a clear framework for configuring the harvester’s potential landscape—demonstrating its versatility for diverse low-frequency applications.

## 5. Conclusions

In this study, a systematic methodology was presented for designing and tuning bistable and multistable mechanical systems by combining elastic buckling with magnetically induced forces. Key design parameters—including the beam’s buckling amplitude $h_{0}$, magnet distance $d_{m}$, and an asymmetry parameter γ—were shown to exert a strong influence on the potential energy landscape, enabling transitions among monostable, bistable, and multistable configurations. An energy-based framework was employed to quantify how these parameters affect well depth, barrier height, and the onset of snap-through dynamics. Additionally, a physics-informed optimization procedure demonstrated that a target force–displacement response can be achieved by judiciously selecting values for these governing parameters. This approach offers a modular concept that can be *programmed* to accommodate a wide range of low-frequency applications, including vibration energy harvesting, vibration isolation, and adaptive structural systems.

Several **limitations** were identified. First, the analysis was restricted to a quasi-static framework, thereby neglecting dynamic phenomena such as transient responses, broadband excitations, and time-varying loads. Second, the effects of damping were not incorporated, although energy dissipation through structural damping or eddy currents in the magnets may alter the stability thresholds and transition dynamics in practical scenarios. Third, deviations arising from manufacturing tolerances and material inconsistencies were not addressed, and these can significantly impact real-world performance. Fourth, the magnetic interaction model used idealized dipole–dipole approximations, ignoring higher-order multipole terms and fringe fields that may become relevant at close proximity. Additionally, scaling remains nontrivial due to the strong nonlinearity and the short-range nature of magnetic forces, complicating both large-scale and highly miniaturized implementations. Finally, although the proposed methodology underscores the potential for reconfigurability, implementing real-time or automated adjustments of magnet positions and polarities remains an open challenge, particularly under practical constraints such as limited space or high operational loads.

Despite these constraints, the findings suggest several **future directions** for advancing this technology. Incorporating dynamic analyses with explicit damping terms and time-dependent forcing will provide a more accurate representation of real operating conditions, facilitating the study of basins of attraction, chaotic orbits, and harvested power at different frequencies. Quantifying stochastic performance indicators is expected to provide industrial level applicability [45]. *Experimental validation* using physical prototypes and high-fidelity computational tools (e.g., Abaqus) is planned to refine theoretical insights and account for geometrical, material, and alignment tolerances. Furthermore, *active control strategies*—such as integrating embedded piezoelectric elements, shape memory alloys, or other smart materials—could introduce adaptive real-time tunability of the stability landscape, thus optimizing energy harvesting performance over a broader range of environmental inputs. Overall, these advancements are expected to significantly enhance the practical viability of multistable harvesters for applications spanning low-power sensor networks, biomedical devices, deployable aerospace structures, and soft robotics.

## Figures and Tables

**Table 1:** Summary of potentials $U^{el}$, $U^{m}$, forces $F^{el}$, $F^{m}$ and their sensitivities with respect to the buckling level $h_{0}$ and magnet distance $d_{m}$.

Operator	Equation Terms			
	$U^{\mathrm{el}}$	$U^{m}$	$F^{\mathrm{el}}$	$F^{m}$
**-**	$\frac{1}{2}k_{1}q^{2}+\frac{1}{4}k_{3}q^{4}$	$-\frac{3\mu_{0}\left(M^{2}h_{A}^{3}h_{B}^{3}\right)}{4\pi \;r^{5}}\;\left[d_{m}-w\left(x,t\right)\right]\;\frac{L}{2}$	$-\left(k_{1}q+k_{3}q^{3}\right)$	$-\frac{3\mu_{0}\left(M^{2}h_{A}^{3}h_{B}^{3}\right)}{4\pi \;r^{4}}\left[1-5\cos^{2}\theta \right]\sin\theta$
** $\frac{\partial }{\partial h_{0}}\left[\cdot \right]$ **	$-\frac{\pi^{4}EAh_{0}}{8L^{3}}\;q^{2}$	0	$-\frac{\pi^{4}EAh_{0}}{4L^{3}}\;q$	0
** $\frac{\partial }{\partial d_{m}}\left[\cdot \right]$ **	0	$-\frac{3\mu_{0}\;L}{8\pi }\;\frac{M^{2}h_{A}^{3}h_{B}^{3}}{r^{5}}\;\times$ $\left[\;1-\frac{5\;{\left(d_{m}-w\left(x,t\right)\right)}^{2}}{r^{2}}\right]$	0	$-\frac{15\mu_{0}\;L}{8\pi }\;\frac{M^{2}h_{A}^{3}h_{B}^{3}}{r^{7}}\;\left(d_{m}-w\left(x,t\right)\right)\;\times$ $\left[-1\;+\;7\;{\left(\frac{d_{m}-w\left(x,t\right)}{r}\right)}^{2}\right]$

**Table 2:** Material and geometric properties of the beam and magnets.

3cBeam Properties			Magnet Properties		
Symbol	Parameter	Value	Symbol	Parameter	Value
*L*	Length	40 mm	$h_{A}$	Height of A	8 mm
*h*	Thickness	0.1 mm	$h_{B}$	Height of B	10 mm
*b*	Width	2 mm	$m_{A}$	Mass	5 g
*E*	Young’s modulus	0.7 GPa	$B_{r}$	Residual Flux Density	1.2 T
ρ	Density	7850 kg/m^3^	$\mu_{0}$	Permeability of free space	$4\pi \times 10^{-7}$ H/m

**Table 3:** Summary of stability mechanisms and their corresponding buckling and magnetic configurations.

Stability Phenomenon	Buckling Involvement ($h_{0}$)	Magnet Configuration (Polarity, $d_{m}$, γ)
**Monostable (1 Well)**		
Linear elastic	No buckling required ($h_{0}=0$)	No magnets required, but can be added for tuning.
**Bistable (2 Wells)**		
Buckling-induced	Requires buckling beyond critical load $P>P_{cr}$. Recommended $h_{0}\approx$ 1–3 mm for practical bistability.	No magnets needed for purely buckling-induced bistability. Can introduce stationary magnets for barrier height tuning.
Magnetically-assisted	Can occur with or without buckling, though combining buckling and magnetic tuning gives better tunability.	Stationary magnets placed symmetrically at $d_{m}$ = 10–30 mm. Magnet polarities aligned for attraction/repulsion depending on tuning objective.
Tunable potential barrier (via $d_{m}$)	Buckling can be present or absent; best tuning occurs when $h_{0}\geq 1.5$ mm.	Magnets placed symmetrically at $d_{m}\approx$ 10–30 mm. Too small $d_{m}$ (<10 mm) leads to low barriers, while too large $d_{m}$ (>50 mm) reduces magnet effects.
Asymmetric bistable	Typically requires buckling $h_{0}\geq 1$ mm for significant asymmetry effects.	Introduced via offset magnets with controlled asymmetry parameter γ. Best results for γ∈[0.5,1.5], with $d_{m}\approx$ 10–20 mm.
**Multistable (3 Wells)**		
Magnetically-induced from monostable	No buckling required; multistability arises purely from magnet forces.	Magnets placed symmetrically at $d_{m}$ = 10–50 mm, with appropriate polarity inversion to generate additional wells.
From bistable + magnet tuning	Requires buckling $h_{0}\geq 1.5$ mm for strong bi-stability effects before transitioning to multistability.	Additional magnets introduced above the beam, with reversed polarity to create new equilibrium wells. Optimal range: $d_{m}$ = 10–30 mm.
Extreme case with barrier height modulation	Typically occurs when $h_{0}\geq 2$ mm, but excessive buckling reduces magnetic influence.	Requires precise tuning of both $d_{m}$ and γ. Best observed for $d_{m}$ = 10–25 mm with moderate asymmetry γ≈1.5.

**Table 4:** Estimated design parameters $\mu_{\mathrm{opt}}\equiv {\left[h_{0},d_{m},\gamma \right]}_{\mathrm{opt}}$ for each configuration to match the target $F_{net}-d$ curve.

$\mu_{\mathrm{opt}}$	Only Magnet	Only Buckling	Magnet + Buckling
$h_{0}$ **(mm)**	-	2.35	2.18
$d_{m}$ **(mm)**	1	-	13.78
γ	1.5	-	1.1

## Data Availability

No new data were created or analyzed in this study. Data sharing is not applicable to this article. The code supporting the conclusions of this article will be made available by the authors on request.
